# Assessment of heat stress contributing factors in the indoor environment among vulnerable populations in Klang Valley using principal component analysis (PCA)

**DOI:** 10.1038/s41598-024-67110-w

**Published:** 2024-07-15

**Authors:** Siti Nurfahirah Muhamad, Vivien How, Fang Lee Lim, Abdah Md Akim, Karmegam Karuppiah, Nur Shabrina Azreen Mohd Shabri

**Affiliations:** 1 Department of Environmental and Occupational Health, Faculty of Medicine and Health Sciences, Universiti Putra Malaysia, Serdang, Selangor Malaysia; 2 Department of Environmental Engineering, Faculty of Engineering and Green Technology (FEGT), Universiti Tunku Abdul Rahman, Kampar, Perak Malaysia; 3 Department of Biomedical Sciences, Faculty of Medicine and Health Sciences, Universiti Putra Malaysia, Serdang, Selangor Malaysia

**Keywords:** Heat stress, Contributing factors, Indoor environment, Vulnerable populations, Principal component analysis (PCA), Climate sciences, Risk factors

## Abstract

Rising global temperatures can lead to heat waves, which in turn can pose health risks to the community. However, a notable gap remains in highlighting the primary contributing factors that amplify heat-health risk among vulnerable populations. This study aims to evaluate the precedence of heat stress contributing factors in urban and rural vulnerable populations living in hot and humid tropical regions. A comparative cross-sectional study was conducted, involving 108 respondents from urban and rural areas in Klang Valley, Malaysia, using a face-to-face interview and a validated questionnaire. Data was analyzed using the principal component analysis, categorizing factors into exposure, sensitivity, and adaptive capacity indicators. In urban areas, five principal components (PCs) explained 64.3% of variability, with primary factors being sensitivity (health morbidity, medicine intake, increased age), adaptive capacity (outdoor occupation type, lack of ceiling, longer residency duration), and exposure (lower ceiling height, increased building age). In rural, five PCs explained 71.5% of variability, with primary factors being exposure (lack of ceiling, high thermal conductivity roof material, increased building age, shorter residency duration), sensitivity (health morbidity, medicine intake, increased age), and adaptive capacity (female, non-smoking, higher BMI). The order of heat-health vulnerability indicators was sensitivity > adaptive capacity > exposure for urban areas, and exposure > sensitivity > adaptive capacity for rural areas. This study demonstrated a different pattern of leading contributors to heat stress between urban and rural vulnerable populations.

## Introduction

Global warming is one of the adverse impacts of climate change, defined as an increment in average global temperatures caused by an increase in the greenhouse effect, which is contributed by increased greenhouse gas emissions^1^. The effects of anthropogenic activities, including industrial processes, agriculture works, waste disposal, and the use of fossil fuel or emission of pollutants, have resulted in elevated surface temperatures, increased extreme weather occurrence, rising sea levels, and fluctuating precipitation patterns. These conditions profoundly impact seven key sectors in Malaysia: public health, agriculture, water resources, biodiversity, forestry, energy, and coastal and marine resources^2,3^. The continuous increment of global temperature trends has the potential to give rise to extreme heat events, commonly known as heat waves^4^.

As defined by the Malaysian Meteorology Department^5^, a daily maximum temperature exceeding 37 °C for three consecutive days is classified as a heat wave. Based on the history of extreme heat occurrence since 2000, the Klang Valley experienced heat waves in 2019 and 2021^6^. Heat waves are prolonged high temperatures that cause significant thermal stress and commonly occur in urban areas with a synergistic effect of urban heat islands (UHI)^7,8^. Due to the cumulative effect of UHI occurrence, heat waves are more severe in cities than in rural areas^7^. Greenhouse emissions may propagate heat waves, and the existing UHI may worsen during the heat wave period, increasing the population's heat stress risk^8^. It is found that the rising surrounding temperature is influenced by higher population density and intensified land use, which are occupied by high-rise and multi-story buildings^9^.

However, heat waves also adversely impact rural communities, particularly on their health. Previous hospital admission and visit data studies found that rural communities commonly recorded higher hospitalization rates during heat waves than urban communities^10^ and the most affected individuals are vulnerable groups such as older people and people with low income^11^. A study of extreme temperature trends across different areas in Klang Valley (2006–2016) found inconsistent results on the annual mean of maximum temperature and the highest value of daily maximum temperature for urban and rural areas^12^. Some rural areas in Klang Valley also reported the same temperature value as urban areas for the annual mean of maximum temperature, which ranges between 31.3 and 31.6 °C, and the highest daily maximum temperature in rural areas ranges between 35.8 and 36.3 °C^12^. This result shows that rural areas in Klang Valley also have the potential to experience high temperatures that exceed 37 °C, where heat waves can occur and affect the rural population, especially vulnerable groups.

The direct heat exposure from the environment commonly measured by the ambient and radiant temperature, relative humidity, and air velocity were significantly related to heat stress^13,14^. However, the intensity of indoor heat exposure may vary depending on other contributing factors, such as building materials, building density, and green spaces^14,15^. Also, the sensitivity of individuals exposed to heat depends on several factors such as age, gender, income, pre-existing diseases, educational level, exercise, and regular smoking^14–18^. Since the vulnerability of heat exposure is also characterized by physical, social, economic, and environmental factors, it could vary significantly within a community and over time. In other words, understanding heat health vulnerability requires more than analysing the direct impacts of a hazard; the concerns about the broader individual, environmental, and socioeconomic conditions that limit people and communities from coping with the effects of a hazard shall also be highlighted.

To date, there is an increasing amount of evidence indicating that both individual and environmental factors contribute to the variability in heat stress experienced by individuals^19–22^, but limited studies were found to emphasize the primary contributing factors influence the heat-health vulnerability, especially among vulnerable population living in urban and rural areas. The current existing heat health mitigation plan prioritizes vulnerable groups but does not sufficiently address area-based concerns. More attention has been given to urban populations subjected to UHI effects^12,23^, with limited investigations into the unique challenges faced by rural populations in managing and adapting to heat stress. Thus, this study aims to determine the precedence of heat stress contributing factors among vulnerable urban and rural populations. This can enhance the understanding of how specific community characteristics in urban and rural settings may influence heat stress differently. Additionally, it highlights the priority on which factors should be given concern in effectively addressing heat-health adaptation and mitigation responses.

## Methods

### Study duration

The study was conducted between May to September 2022, coinciding with the Southwest monsoon period in Malaysia. The Southwest monsoon in Malaysia typically spans from May to September and is characterized by low precipitation, less cloud cover, high outgoing long-wave radiation, often featuring a dry period^5^. This study focused on this period due to its reputation for elevated temperatures, as previously classified as the hottest months of the year (April, May, and June) in prior research^6^. It is assumed that the temperatures during the Southwest monsoon are indicative of heatwaves conditions. This study involves three phases: Phase I: the pilot study; Phase II: screening; and Phase III: data collection for the main study. A pilot study involving 20 randomly selected participants was conducted in June 2022 to obtain the prevalence of heat stress symptoms, followed by the screening phase in July 2022 and data collection for the main study between July and September 2022.

### Study participants

A comparative cross-sectional study design was used, and an equal number of respondents from urban and rural areas in the Klang Valley, Malaysia, were recruited using stratified random sampling. The calculation of the sample size using comparing two means formula^24^ was based on the prevalence of heat stress symptoms reported in the pilot study for both urban and rural areas. A total of 108 respondents were recruited, achieving a 96% response rate. The study participants were selected through a screening checklist based on the inclusive criteria. Those who were classified as vulnerable in any groups (aged 60 years old and above, people with health morbidity, and people with low income) and experiencing any heat stress symptoms for the preceding months (April to June) while residing in their residential areas were included in this study. Conversely, pregnant women and individuals under 13 years old were excluded from this study due to hormonal, blood volume and circulation changes in pregnant women^25^ as well as the underdevelopment of thermoregulatory mechanisms in children^26,27^, which have the potential to confound the study results.

### Research tools

An adapted questionnaire from the Guidelines on Heat Stress Management at Workplace^13^ was used to assess heat stress symptoms during Phases I and II. In Phase III, a self-administered questionnaire was used to obtain the participants' sociodemographic background, health status, lifestyle information, and residential information for the target population. The questionnaire underwent content validation, achieving a Scale Content Validity Index (S-CVI) value of 1.0, which is considered acceptable^28^. A pre-test was conducted during the pilot study to assess test–retest reliability. The intraclass correlation coefficient for the continuous data ranged from 0.78 and 0.99, indicating good reliability (0.75–0.9), and excellent reliability (> 0.9)^29^. Cohen’s Kappa coefficient for nominal data ranged from 0.89 to 1.00, indicating almost perfect agreement^30^. For lifestyle information, we adapted International Physical Activity Questionnaire^31^, and physical activity levels were classified using metabolic equivalent of task (MET) value, obtained from MET score calculation (combination of physical intensity (walking, moderate-intensity, and vigorous-intensity) and hours spend on physical activity by the respondents)^32^. The relevant questions for each section are provided in Table 1.

**Table 1:** Variables included in the PCA analysis.

Type of factor	Factors	Indicators (for + loadings value)
Sociodemographic factors	Age	Sensitivity
	Gender (Female)	Adaptive capacity
	Body mass index (BMI)	Adaptive capacity
	Household income	Adaptive capacity
	Educational level	Adaptive capacity
Health factors	Health morbidity (Yes)	Sensitivity
	Medicine intake (Yes)	Sensitivity
Lifestyle factor	Smoking (Yes)	Adaptive capacity
	Daily water intake	Adaptive capacity
	Physical activity level	Adaptive capacity
	Occupation type (outdoor work)	Adaptive capacity
Residential factors	Building age	Adaptive capacity
	Building type (multi-story building)	Exposure
	Building density	Exposure
	Roof material (high thermal conductivity)	Exposure
	Wall material (high thermal conductivity)	Exposure
	Building size	Adaptive capacity
	Green plot ratio	Adaptive capacity
	Ceiling availability (Yes)	Adaptive capacity
	Ceiling/ roof height	Adaptive capacity
	Residency duration	Adaptive capacity

### Ethics approval

This study obtained ethical approval from Ethics Committee for Research Involving Human Subjects, Universiti Putra Malaysia (Reference No: JKEUPM-2022-222). All data collection methods were performed in accordance with the respective ethical guidelines. Written informed consent was obtained from participants and parents or guardians of minors before proceeding with the data collection.

### Statistical analysis

The data obtained from the respondents were analyzed using Statistical Package for the Social Sciences (SPSS) version 25. A descriptive analysis was used to get the average and frequency of sociodemographic background, health status, lifestyle information, and residential information. A principal component analysis (PCA) was conducted to determine the factors contributing to heat stress. Data preprocessing, which included the encoding categorical variables and the standardizing numerical variables, was applied before the PCA analysis^33^. PCA for mixed data was employed, leveraging its powerful technique in interpreting the status of variables across different data types^34^. Only datasets with a Kaiser–Meyer–Olkin measure (KMO) value of > 0.5^35^ and Barlett’s test of sphericity (BTS) yielding a result of 0.000 (*p* < 0.001) were selected for interpretation^33^.

PCA was used for the data reduction by extracting a limited number of principal components (PCs), and varimax rotation was applied to maximize the variances of factor loadings across variables of each factor to enhance the interpretability of the result^36^. The PC variables with a factor loading of 0.4 and higher were selected as significantly loaded items as recommended for the rotated factor pattern^36^. Principal components (PCs) with eigenvalue > 1.0 were extracted for the result. A cumulative variance of at least 60% is considered acceptable^35^. Additionally, PCs with a percentage of more than 10% variance were highlighted as the main contributing factors of heat stress and were further categorized into three heat health vulnerability indicators, which are sensitivity, exposure and adaptability based on the variables' criteria^37^. Table 1 shows the variables included in the PCA analysis.

## Results

### Sociodemographic background, health status, lifestyle information and residential information

Table 2 shows the sociodemographic background of the study population from urban and rural areas. A total of 108 Malaysians aged 13 years old and above were recruited in this study; 54 participants were from urban areas, and 54 were from rural areas. In urban areas, the average age of the study population is 53 ± 11.4 years old, with age range of 24 to 74 years old. Most of the respondents from urban areas are female (85.2%). The average body mass index (BMI) is 28.9 ± 6.99 kg/m^2^, classified as overweight by the Ministry of Health Malaysia^38^. The highest education level among the study population was recorded for secondary school (68.5%), followed by no formal education (13.0%) and primary school (11.1%). Most respondents are involved in indoor-type of occupations (92.6%) such as cleaning sectors, working from home, office clerk, lorry driver, and housewife. Notably, 90.7% of the respondents were from low-income or B40 groups (which represent the bottom 40% of income in Malaysia, according to the Department of Statistics Malaysia^39^.

**Table 2:** Sociodemographic background of the study population (N = 108).

Sociodemographic characteristics	Urban (n = 54)	Rural (n = 54)
	Mean (SD)	
Continuous variables		
Age (years)	52.8 (11.44)	49.4 (16.1)
Body mass index, BMI (kg/m^2^)	28.9 (6.99)	27.2 (6.42)
Duration of residency (years)	16.6 (6.34)	30.4 (6.52)
**Categorical variables**	**Frequency, *f* (%)**	
Age groups		
< 60 years old	39 (72.2)	38 (70.4)
≥ 60 years old	15 (27.8)	16 (29.6)
*Gender*		
Male	8 (14.8)	18 (33.3)
Female	46 (85.2)	36 (66.7)
*Educational level*		
No formal education	7 (13.0)	1 (1.9)
Primary school	6 (11.1)	16 (29.6)
Secondary school	37 (68.5)	27 (50.0)
Tertiary education (diploma/undergraduate/graduate)	4 (7.4)	10 (18.5)
*Household income (Ringgit Malaysia, RM)*		
B40 (Below RM4851)	49 (90.7)	48 (88.9)
M40 (RM4851-RM10,970)	5 (9.3)	6 (11.1)
Occupation type		
Indoor work	50 (92.6)	47 (87.0)
Outdoor work	4 (7.4)	7 (13.0)

For rural areas, the average age of the study population is 49 ± 16.1 years old, with an age range of 13 to 76 years old. The majority of participants from rural areas are female (66.7%). The average body mass index (BMI) of respondents from rural areas is 27.2 ± 6.42 kg/m^2^, which also falls in the overweight category. Most of the respondents from rural areas had the highest education level of secondary school (50.0%), followed by primary school (29.6%), and tertiary education (18.5%). Most of them are involved in indoor-type occupations (87.0%) compared to outdoor-type (13.0%), where the typical outdoor jobs among them are agriculture-based, such as farmer, gardener, and landscaper. It was recorded that 88.9% of the respondents were categorized as low-income or B40 groups.

Table 3 shows the health status and lifestyle information of the study population. Based on the result, 46.3% of the urban area respondents and 53.7% of the rural areas respondents have health morbidities such as hypertension, diabetes, obesity, respiratory diseases, cardiovascular diseases, kidney diseases, and skin diseases. More rural respondents (48.1%) were taking medicine regularly as prescribed by medical practitioners compared to the urban respondents (38.9%). The average daily water intake for urban and rural respondents was within the recommended amount by the Ministry of Health Malaysia^40^, ranging between 1.5 and 2.0 L per day. The calculated MET value categorizes most urban (53.7%) and rural (46.3%) respondents being at a moderate level of physical activity. Only 3.7% of urban and 16.7% of rural respondents smoke. None of the respondents is regular alcohol drinkers.

**Table 3:** Health status and lifestyle information of the study population (N = 108).

Health status and lifestyle variables	Urban (n = 54)	Rural (n = 54)
	Mean (SD)	
Continuous variables		
Daily water intake (litre)	1.7 (0.8)	1.6 (0.7)
**Categorical variables**	**Frequency, *f* (%)**	
*Health morbidity*		
Yes	25 (46.3)	29 (53.7)
No	29 (53.7)	25 (46.3)
*Medicine intake (prescribed by a medical practitioner)*		
Yes	21 (38.9)	26 (48.1)
No	33 (61.1)	28 (51.9)
*Physical activity level (based on MET value)*		
Low	10 (18.5)	8 (14.8)
Moderate	29 (53.7)	25 (46.3)
High	15 (27.8)	21 (38.9)
Smoking		
Yes	2 (3.7)	9 (16.7)
No	52 (96.3)	45 (83.3)
*Regular alcohol intake (at least once daily)*		
Yes	0 (0.0)	0 (0.0)
No	54 (100.0)	54 (100.0)

Table 4 shows the residential information of the study population. Most urban respondents (83.3%) live in multi-story buildings, staying for almost 17 ± 6.34 years. In contrast, most rural respondents (61.1%) live in landed houses, staying for nearly 30 ± 6.52 years. For the residential information, the average building age (year) in urban areas is 26.41 ± 10.18 and 29.70 ± 10.29 for rural. The building size (m^2^) is relatively lower in urban areas (80.67 ± 25.37) compared to rural areas (165.82 ± 73.6). Most urban (88.9%) and rural (61.1%) houses equipped with ceilings, with an average of 3.51 ± 0.63 m and 4.26 ± 1.38 m in height, respectively. The wall material for urban residential buildings is primarily concrete (83.3%), whereas in rural areas primarily bricks with cement plaster (63.0%). Both urban and rural houses mainly used concrete as roof material. Building density and green plot ratios recorded in urban and rural areas showed that urban areas are denser with buildings and lower with green spaces than rural areas.

**Table 4:** Residential information of the study population (N = 108).

Residential Information	Urban (n = 54)	Rural (n = 54)
	Mean (SD)	
Continuous variables		
Building age (year)	26.41 (10.18)	29.70 (10.29)
Building size (m^2^)	80.67 (25.37)	165.82 (73.6)
Ceiling/ roof height (m)	3.51 (0.63)	4.26 (1.38)
Building density (%)	23.15 (3.14)	16.87 (5.22)
Green plot ratio (%)	18.73 (8.16)	47.66 (14.20)
Residency duration (year)	16.6 (6.34)	30.4 (6.52)
**Categorical variables**	**Frequency, *f* (%)**	
*Building type*		
Landed house	9 (16.7)	33 (61.1)
Multi-story building	45 (83.3)	21 (38.9)
Wall material		
Brick with plaster	9 (16.7)	34 (63.0)
Concrete	45 (83.3)	20 (37.0)
Roof material		
Ceramic/clay roof tiles	2 (3.7)	14 (25.9)
Concrete roof tiles/concrete slab	49 (90.7)	22 (40.7)
Zinc	3 (5.6)	18 (33.3)
*Ceiling availability*		
Yes	48 (88.9)	33 (61.1)
No	6 (11.1)	21 (38.9)

Green plot ratio (%) = percentage of green spaces in 16000 m^2^ land area.

Building density (%) = percentage of building density in 16000 m^2^ land area.

### Heat stress contributing factors

Table 5 shows the result of PCA for the urban vulnerable population. Based on PCA analysis for urban areas, 14 factors were identified as contributing factors with significant loadings value (≥ 0.4), further grouped into five principal components (PCs). The five PCs with eigenvalue > 1.0 explain 64.3% of the variability from the original contributing factors. The components comprised PC1 (health morbidity [yes], medicine intake [yes], and increased age), PC2 (occupation type [outdoor], ceiling availability [no], and longer residency duration), PC3 (lower ceiling height and increased building age), PC4 (lower BMI, higher educational level and gender [male]), and PC5 (smoking [yes], lower daily water intake, and lower physical activity level).

**Table 5:** Principal component analysis (PCA) for urban vulnerable population (n = 54).

	Components				
	1	2	3	4	5
Eigenvalue	2.575	2.112	1.606	1.372	1.34
% of Variances	**18.396**	**15.087**	**11.469**	9.801	9.572
Cumulative %	18.396	33.483	44.951	54.752	64.324
	Rotated factor pattern: Varimax rotation method				
*Variables*					
Health morbidity	**0.855**	−0.047	0.274	−0.002	0.025
Medicine intake	**0.848**	0.002	0.26	0.054	0.062
Age	**0.675**	0.133	−0.258	−0.152	0.001
Occupation type	0.029	**0.828**	−0.081	0.118	−0.097
Ceiling availability	0.111	**−0.742**	−0.057	0.325	−0.02
Residency duration	0.198	**0.683**	0.268	0.183	−0.12
Ceiling height	−0.253	−0.017	**−0.749**	−0.132	0.332
Building age	0.018	0.105	**0.712**	−0.219	0.211
Body mass index (BMI)	0.021	0.068	0.058	**−0.757**	0.146
Educational level	−0.459	0.041	0.381	**0.522**	0.07
Gender	0.066	-0.407	0.287	**−0.516**	−0.174
Smoking	0.03	-0.015	−0.045	0.305	**0.816**
Daily water intake (liter)	−0.188	0.072	−0.208	0.284	**−0.594**
Physical activity level	0.125	0.107	0.184	0.316	**−0.485**

Variable is significantly loaded at ≥ 0.4.

Extraction Method: Principal Component Analysis (Eigenvalue > 1.0).

Rotation Method: Varimax with Kaiser Normalization.

KMO value: 0.504, Bartlett’s test: *p*  < 0.001.

Table 6 shows the result of PCA for the rural vulnerable population. Based on PCA analysis for rural areas, 15 factors were identified as contributing factors with significant loading values ≥ 0.4), further grouped into five PCs. The five PCs with eigenvalue > 1.0 explain 71.5% of the variability from the original contributing factors. The components comprised PC1 (ceiling availability [no], higher thermal conductivity roof material, increased building age, shorter residency duration), PC2 (health morbidity [yes], medicine intake [yes], and increased age), PC3 (gender [female], higher BMI, and smoking [no]), PC4 (increased household income, higher educational level, lower daily water intake), and PC5 (higher physical activity level and occupation type [outdoor]).

**Table 6:** Principal component analysis (PCA) for rural vulnerable population (n = 54).

	Components				
	1	2	3	4	5
Eigenvalue	3.839	2.538	1.966	1.318	1.063
% of Variances	**25.594**	**16.922**	**13.105**	8.789	7.085
Cumulative %	25.594	42.516	55.621	64.41	71.494
	Rotated factor pattern: Varimax rotation method				
*Variables*					
Ceiling availability	**−0.942**	−0.138	0.046	−0.055	−0.041
Roof material	**0.936**	−0.103	0.088	−0.072	−0.056
Building age	**0.915**	0.162	−0.155	0.147	−0.003
Residency duration (years)	**−0.626**	0.001	0.157	−0.064	−0.177
Health morbidity	0.062	**0.928**	0.003	−0.046	−0.103
Medicine intake	0.173	**0.925**	0.163	−0.006	−0.07
Age	0.469	**0.526**	0.088	−0.131	0.223
Gender	−0.065	0.297	**0.825**	0.141	−0.145
Body mass index (BMI)	0.167	0.042	**0.728**	−0.14	−0.043
Smoking	0.181	0.047	**−0.627**	−0.329	0.04
Household income	0.032	−0.003	0.135	**0.758**	0.076
Educational level	0.086	−0.244	0.158	**0.757**	−0.279
Daily water intake (litre)	−0.083	−0.129	0.441	**−0.567**	−0.069
Physical activity level	−0.187	−0.046	−0.009	0.072	**0.868**
Occupation type	0.25	−0.075	−0.331	-0.211	**0.587**

Variable is significantly loaded at ≥ 0.4.

Extraction Method: Principal Component Analysis (Eigenvalue > 1.0).

Rotation Method: Varimax with Kaiser Normalization.

KMO value: 0.648, Bartlett’s test: *p* < 0.001.

Urban and rural areas have three PCs with more than 10% variances, highlighted as the primary contributing factors to heat stress. For urban, PC1 (health morbidity, medicine intake, and increased age) can be classified as sensitivity, PC2 (outdoor occupation type, lack of ceiling, and longer residency duration) as adaptive capacity, and PC3 (lower ceiling height and increased building age) as exposure. For rural areas, PC1 (lack of ceiling, higher thermal conductivity roof material, increased building age, and shorter residency duration) can be classified as exposure, PC2 (health morbidity, medicine intake, and increased age) as sensitivity, and PC3 (female, non-smoking, and increased BMI) as adaptive capacity. To summarize, the pattern of heat-health vulnerability indicators in urban areas in decreasing order according to the percentage of variances was sensitivity > adaptive capacity > exposure. In contrast, heat-health vulnerability indicators in rural areas were exposure > sensitivity > adaptive capacity.

## Discussion

Based on the results, different patterns of heat stress-contributing factors were discovered between urban and rural areas in this study. This finding corresponded to a previous study that agreed heat stress-contributing factors varied in different areas^41^. A comparative study on the risk factors of heat related illnesses between urban and rural areas found that the urban population is influenced by low education levels, poverty, living in old building structures, and mobile homes with poor insulation. In contrast, risk factors in rural areas include elderly, outdoor workers in agricultural sectors, mobile homes with poor insulation, and developed land^42^. Another study indicated that urban populations are more susceptible to heat stress due to high heat-prone areas, while rural populations influenced by poor health status, poverty, and challenges in accessing healthcare related to geographic and finances^43^. While other studies classified heat stress contributing factors into several groups, commonly individual, environmental, and occupational^44,45^, our study categorizes these factors into three heat health vulnerability indicators, which are sensitivity, exposure, and adaptive capacity to enhance the appropriate mitigation measures during extreme heat events.

This study revealed that sensitivity is the most impactful indicator influencing heat-health vulnerability in urban areas (PC1), while it ranks as a second most important indicator in rural areas (PC2). Both areas highlighted similar factors, including the presence of health morbidity, medicine intake, and older age. Individuals with health morbidities such as diabetes, obesity, hypertension, respiratory disease, and cardiovascular disease have physiological deficiencies for acclimatization^46^, which potentially influence the relationship between heat exposure and adverse health impacts^47^. Additionally, medication consumption can interfere with thermoregulation, as anticholinergics can lead to dehydration^48^. Increased age has been linked with reduced sweat output, which is associated with a decrease in epidermal blood flow during heat exposure, thereby reducing the body’s ability for thermoregulation^48^. Previous studies have also agreed that poor health status and elderly are factors related to sensitivity, increasing heat-related health issues among urban populations^49–51^, and rural populations^52,53^.

Exposure was found to be the most impactful indicator in rural areas (PC1), while it falls in third priority in urban areas (PC3). Most of the factors listed in PCs for both areas are related to residential characteristics. However, increased building age is found to be a similar factor in both areas. Other highlighted factors in rural areas include higher thermal conductivity roof material, absence of ceiling installation, and reduced residency duration, while lower ceiling height is highlighted as exposure factor in urban areas. Other studies have also outlined residential characteristics as exposure indicator in urban^53^, and rural areas^54^. Most of the previous studies highlighted direct sources of heat such as land surface temperature^50,54^, heatwave occurrence^55^, and high ambient temperature^56^ as exposure indicator. Nonetheless, some studies have also classified contributing factors such as housing characteristics as exposure indicator in urban^53^ and rural areas^52^. Previous studies have agreed that the outdoor-indoor temperature varies depending on the building characteristics^57,58^.

Adaptive capacity is another indicator ranked as the second most impactful indicator in urban areas (PC2) and third (PC3) in rural areas. However, distinct factors were observed in both PCs. Factors influencing heat stress in urban areas consist of outdoor occupation type, absence of ceiling installation, and longer residency duration, all of which may expose individuals to higher heat exposure. However, long-term exposure to hot conditions may lead to acclimatization, contributing to a better adaptive mechanism^59,60^. In contrast, rural areas highlighted gender (female), higher BMI, and non-smoking as components of adaptive capacity. Females may tolerate heat more efficiently than males due to a broader range of their resting core temperature, although they have a slower sweating response compared to males^61^. Although it is common for higher BMI to increase insulation and heat retention, it may also serve as a reservoir for fluid and electrolytes, aiding in thermoregulation and maintaining hydration status during heat exposure^62^. While other studies have outlined residential and environmental-related factors such as building type^63^ and green spaces or vegetation index^64,65^ as components of adaptive capacity, our study proposes acclimatization-induced factors (urban) and individual factors (rural) as components of adaptive capacity.

Our study addresses previous research gaps in several ways. Firstly, this study emphasizes comparing the patterns of heat stress contributing factors between urban and rural areas. Secondly, our study focuses on vulnerable populations to enhance understanding of heat health vulnerability, as these groups are often linked to high morbidity and mortality rates. Although prior studies have provided information on heat stress contributing factors, limited emphasis has been placed on highlighting the precedence of these factors. Furthermore, existing mitigation plans lack area-based concerns. Therefore, our findings are essential for providing baseline information to address appropriate heat mitigation measures based on priority and specific areas or populations.

However, it is important to address the study’s limitations. While acknowledging that a heatwave is defined by prolonged abnormally high temperatures, this study does not equate temperatures recorded during the Southwest monsoon with heat waves. Instead, we assumed that the sampling periods could reflect heat wave conditions based on the climate data mentioned in the previous study, rather than conducting heat exposure monitoring to confirm the existence of extreme heat or heat wave occurrence. Future research endeavors could explore the specific quantification of heatwave events during the Southwest monsoon period to further refine the understanding of heat stress dynamics in this region and enhance the validity of these findings. Additionally, it is worth noting that data collection was primarily conducted on weekdays, which may have limited the randomization of respondents, particularly concerning working adults and young adults attending school.

## Conclusion

This study demonstrates different patterns of primary heat stress contributors among vulnerable populations in urban and rural areas. It sheds light on the unique challenges faced by both urban and rural vulnerable populations. The findings not only enhance our understanding of how specific community characteristics in urban and rural settings may influence heat stress differently, but also highlight the factors that should be prioritized to effectively address appropriate heat health adaptation and mitigation responses, reducing the adverse impacts of excessive heat exposure on vulnerable populations.

## Data Availability

The datasets used and/or analyzed in this study available from the corresponding author on reasonable request.
