# Author Correction: Assessment of heat stress contributing factors in the indoor environment among vulnerable populations in Klang Valley using principal component analysis (PCA)

**DOI:** 10.1038/s41598-024-75132-7

**Published:** 2024-10-14

**Authors:** Siti Nurfahirah Muhamad, Vivien How, Fang Lee Lim, Abdah Md Akim, Karmegam Karuppiah, Nur Shabrina Azreen Mohd Shabri

**Affiliations:** 1 Department of Environmental and Occupational Health, Faculty of Medicine and Health Sciences, Universiti Putra Malaysia, Serdang, Selangor Malaysia; 2 Department of Environmental Engineering, Faculty of Engineering and Green Technology (FEGT), Universiti Tunku Abdul Rahman, Kampar, Perak Malaysia; 3 Department of Biomedical Sciences, Faculty of Medicine and Health Sciences, Universiti Putra Malaysia, Serdang, Selangor Malaysia

Correction to: *Scientific Reports*10.1038/s41598-024-67110-w, published on 15 July 2024

The original version of this Article contained an error in the Acknowledgements section.

“The authors would like to thank the Fundamental Research Grant Scheme (FRGS), Ministry of Science Technology and Innovation (MOSTI), Malaysia (Vote No. 5540402) for the financial support.”

now reads:

“This work was funded by the Higher Education, Malaysia (MOHE) under the Fundamental Research Grant Scheme (FRGS/1/2020/SKK06/UPM/02/1)”

The original Article has been corrected.

